# Supplementary material for: HIVprotI: an integrated web based platform for prediction and design of HIV proteins inhibitors
Source: J Cheminform. 2018 Mar 9;10:12. doi: 10.1186/s13321-018-0266-y (PMC5845081; doi:10.1186/s13321-018-0266-y)
Supplement: Supplementary file 2 — Additional file 2. Source code of HIVProtI web server. [file 13321_2018_266_MOESM2_ESM.zip › HIVprotI_Source-code/hivprotI/weka-3-6-6/doc/weka/gui/MainMenuExtension.html]

MainMenuExtension


---


|  |  |  |
| --- | --- | --- |
| **Overview** \| **Package** \| **Class** \| **Tree** \| **Deprecated** \| **Index** \| **Help** \| **Weka's home** | |  |
| **PREV CLASS**   **NEXT CLASS** | **FRAMES**    **NO FRAMES**     **All Classes** |  |
| SUMMARY: NESTED \| FIELD \| CONSTR \| METHOD | DETAIL: FIELD \| CONSTR \| METHOD |  |


---


## weka.gui Interface MainMenuExtension

---

``` public interface MainMenuExtension ```

Classes implementing this interface will be displayed in the "Extensions"
menu in the main GUI of Weka.

**Version:**
:   $Revision: 1.1 $

**Author:**
:   fracpete (fracpete at waikato dot ac dot nz)

---

| **Method Summary** | |
| --- | --- |
| `void` | `fillFrame(java.awt.Component frame)`             Fills the frame with life, like adding components, window listeners, setting size, location, etc. |
| `java.awt.event.ActionListener` | `getActionListener(javax.swing.JFrame owner)`             If the extension has a custom ActionListener for the menu item, then it must be returned here. |
| `java.lang.String` | `getMenuTitle()`             Returns the name of the menu item. |
| `java.lang.String` | `getSubmenuTitle()`             Returns the name of the submenu. |

**Method Detail**

### getSubmenuTitle

```
java.lang.String getSubmenuTitle()
```

:   Returns the name of the submenu. If there is no submenu necessary then
    the return value is null.

    :   **Returns:**: the title of the submenu or null if no submenu

---


### getMenuTitle

```
java.lang.String getMenuTitle()
```

:   Returns the name of the menu item.

    :   **Returns:**: the name of the menu item.

---


### getActionListener

```
java.awt.event.ActionListener getActionListener(javax.swing.JFrame owner)
```

:   If the extension has a custom ActionListener for the menu item, then it
    must be returned here. Having a custom `ActionListener` also
    means that the component handles any frame by itself.

    :   **Parameters:**: `owner` - the owner of potential dialogs **Returns:**: a custom ActionListener, can be null **See Also:**: `fillFrame(Component)`

---


### fillFrame

```
void fillFrame(java.awt.Component frame)
```

:   Fills the frame with life, like adding components, window listeners,
    setting size, location, etc. The frame object can be either derived from
    `JFrame` or from `JInternalFrame`. This method is
    only called in case `getActionListener()` returns null.

    :   **Parameters:**: `frame` - the frame object to embed components, etc. **See Also:**: `getActionListener(JFrame)`, `JFrame`, `JInternalFrame`


---


|  |  |  |
| --- | --- | --- |
| **Overview** \| **Package** \| **Class** \| **Tree** \| **Deprecated** \| **Index** \| **Help** \| **Weka's home** | |  |
| **PREV CLASS**   **NEXT CLASS** | **FRAMES**    **NO FRAMES**     **All Classes** |  |
| SUMMARY: NESTED \| FIELD \| CONSTR \| METHOD | DETAIL: FIELD \| CONSTR \| METHOD |  |


---
